# Ferroptosis: mechanism, immunotherapy and role in ovarian cancer

**DOI:** 10.3389/fimmu.2024.1410018

**Published:** 2024-08-13

**Authors:** Ke Guo, Miao Lu, Jianlei Bi, Tianyu Yao, Jian Gao, Fang Ren, Liancheng Zhu

**Affiliations:** ^1^ Department of Obstetrics and Gynecology, Shengjing Hospital of China Medical University, Shenyang, Liaoning, China; ^2^ Department of Obstetrics and Gynecology, The Second Hospital of Dalian Medical University, Dalian, Liaoning, China

**Keywords:** ferroptosis, ovarian cancer, Xc system, MDSC, immunotherapy

## Abstract

Ovarian cancer is currently the second most common malignant tumor among gynecological cancers worldwide, primarily due to challenges in early diagnosis, high recurrence rates, and resistance to existing treatments. Current therapeutic options are inadequate for addressing the needs of ovarian cancer patients. Ferroptosis, a novel form of regulated cell death with demonstrated tumor-suppressive properties, has gained increasing attention in ovarian malignancy research. A growing body of evidence suggests that ferroptosis plays a significant role in the onset, progression, and incidence of ovarian cancer. Additionally, it has been found that immunotherapy, an emerging frontier in tumor treatment, synergizes with ferroptosis in the context of ovarian cancer. Consequently, ferroptosis is likely to become a critical target in the treatment of ovarian cancer.

## 1 Introduction

Ovarian cancer is one of the most prevalent and deadly subtypes of gynecological malignant tumors. There are several types of ovarian cancer, with epithelial ovarian cancer being the most common (1). Ovarian cancer is typically diagnosed using a combination of therapies, including surgery, chemotherapy, and innovative immunotherapy. Between 1976 and 2015, the death rate from ovarian cancer in the United States declined by 33%, while its incidence fell by 29% between 1985 and 2014 (2). However, the five-year survival rate of patients remained less than thirty percent (2), leading to a higher mortality rate among female reproductive system malignancies, as the majority of ovarian cancers were only discovered in stage III (51%) or stage IV (29%) (3). Furthermore, there is a lack of treatment resistance and early diagnostic targets for ovarian cancer. Therefore, novel therapeutics are urgently needed to improve the early diagnosis, treatment, and prognosis of ovarian cancer.

Ferroptosis is a novel process of iron-dependent regulated cell death induced by erastin, characterized by the accumulation of iron ions, increased lipid peroxide concentration, reduced glutathione peroxidase 4 (GPx4) activity, and often accompanied by large amounts of reactive oxygen species (4). Ferroptosis has been shown to be intimately associated with several biological processes and diseases, including Alzheimer’s disease and brain hemorrhage. Furthermore, it has been proposed that ferroptosis may play a role in tumor suppression (5). Recent studies have demonstrated that ferroptosis is closely related to the growth regulation of ovarian cancers through mechanisms involving the transsulfuration pathway, Hippo signaling pathways, and p53. Additionally, the combination of immunotherapy and ferroptosis treatment is becoming a research hotspot in ovarian cancer. This review outlines the current understanding and research on ferroptosis in ovarian cancer, as many specific regulatory processes and mechanisms remain unclear. Further elucidation of the ferroptosis process in ovarian cancer is expected to identify more therapeutic targets and drugs, laying the groundwork for novel treatment approaches and improved prognoses.

## 2 Mechanism of ferroptosis

**Figure 1:**
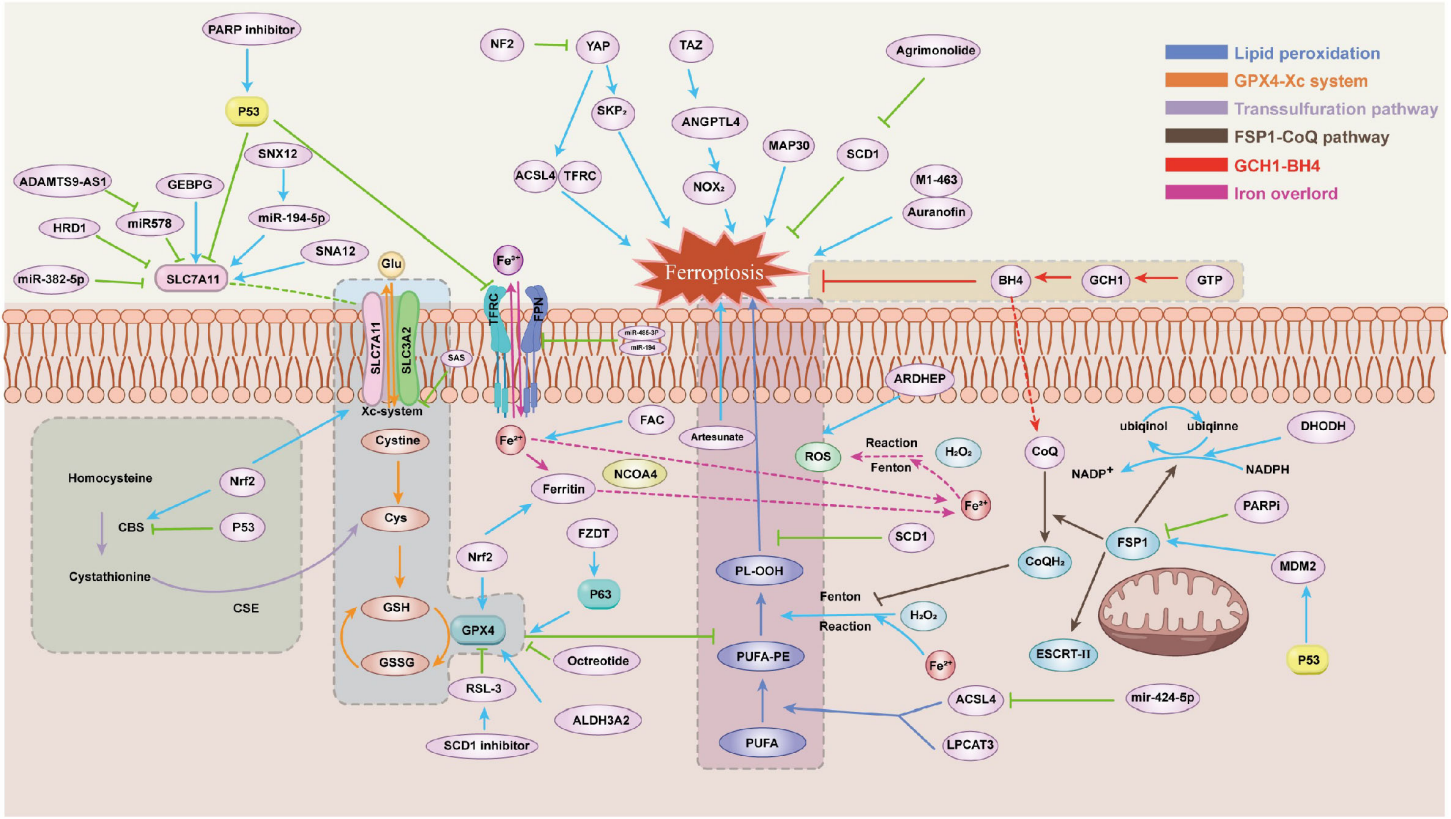
The main mechanism of ferroptosis.

Ferroptosis is commonly believed to be regulated primarily by three mechanisms: iron overload, lipid peroxidation, and the oxidized form mediated by the xc-cysteine/glutamate antiporter system (6).Notably, ferroptosis is primarily caused by unbalanced cellular metabolic processes, including dysregulated iron and lipid metabolism, and the production of reactive oxygen species (ROS) (7).This unique characteristic of ferroptosis has led to the discovery of new pathways and mechanisms which closely related to ferroptosis regulation in recent years (**Figure 1**).

### 2.1 GPX4-GSH pathway

The XC-system is an amino acid transporter that forms the GPX4-GSH pathway with the functional subunit SLC7A11 and the regulatory subunit SLC3A2, playing a crucial role in the cellular antioxidant system. Through the XC-system, cystine and glutamate are exchanged between the inside and outside of the cell. Inside the cell, cystine is converted to cysteine, which is further transformed into glutathione (GSH). GSH acts as a cofactor for glutathione peroxidase 4 (GPX4), helping to restore polyunsaturated fatty acids (PUFAs) and inhibiting ferroptosis, making it a vital regulator of ferroptosis (8). When GPX4 is suppressed, it promotes the accumulation of lipid ROS, accelerates cell death, induces the occurrence of ferroptosis, and inhibits the growth of tumor cells (9). Studies have found that drugs such as erastin (10), sulfasalazine (11), ssorafenib (12), and p53 (13)can causing the production of GSH by inhibiting the XC system and induce the occurrence of ferroptosis. In ovarian cancer, Luo et al. discovered that PAX8, a gene that suppresses ferroptosis, can be inhibited by PAX8i to induce ferroptosis through the GPX4 pathway in combination with RSL3. This approach increases sensitivity to ferroptosis inducers and inhibits the progression of ovarian cancer (14).

Mammal cells can also obtain cysteine through the transsulfuration pathway in certain cases, in addition to their dependence on the XC system (15). Methionine can be converted to cysteine via the catalytic actions of cystathionine β-synthase (CBS) and cystathionine gamma-lyase (CSE). Verschoor and colleagues discovered that blocking the transsulfuration pathway lowers the level of GSH and GPX activity in an ovarian cancer model cell line (16), which ultimately results in ferroptosis.

### 2.2 Lipid peroxidation

Ferroptosis is primarily caused by the lipid peroxidation of polyunsaturated fatty acids (PUFAs). The acyl-CoA synthetase long-chain family member 4 (ACSL4) and lysophosphatidylcholine acyltransferase 3 (LPCAT3) catalyze the production of polyunsaturated fatty acid-phosphatidylethanolamine (PUFA-PE) from PUFA (17). PUFA-PE is the most susceptible to the oxidation of lipids, which under the influence of the Fenton reaction undergo a series of oxidative reactions, thereby promoting the production of PL-OOH (18).This extensive lipid peroxide reaction induces the occurrence of ferroptosis (19). Sebastian Doll (20) and colleagues found that inhibiting ACSL4 can suppress ferroptosis by regulating lipid peroxidation, suggesting that ACSL4 may be a potential target for preventing ferroptosis-related diseases.

### 2.3 FSP1-CoQ10-NAD (P) H pathway

A recently identified antioxidant system that controls ferroptosis without relying on the GPX4 pathway is ferroptosis suppressor protein 1 (FSP1) (21). By reducing and restoring coenzyme Q10 (CoQ10), which inhibits lipid peroxide, FSP1 is drawn to the plasma membrane to prevent ferroptosis. FSP1, possessing NAD(P)H oxidase activity (22), can catalyze the conversion of ubiquinone to ubiquinol, with the resulting ubiquinol scavenging free radicals and thereby preventing ferroptosis. However, Kang et al. discovered that in FSP1-knockout cells, CoQ10 cannot reverse the ferroptosis induced by ferroptosis inducers. On the other hand, the ESCRT-III membrane repair system lets the ferroptosis inductor do its job again, which is to cause ferroptosis in FSP-1 cells. Consequently, it is unlikely that FSP1 might also be involved in ESCRT-III-mediated ferroptosis suppression (23). Additionally, it has been shown that dihydroorotate dehydrogenase (DHODH) can prevent ferroptosis in the mitochondrial membrane by converting ubiquinone to ubiquinol. In GPX4-deficient cancer cells, DHODH inactivation leads to extensive lipid peroxidation and ferroptosis (24). However, none of the specific mechanisms are clear enough.

### 2.4 The BH4-GCH1 pathway

GTP cyclohydrolase 1 (GCH1) produces tetrahydrobiopterin (BH4) from GTP, acting as a speed limit enzyme. GCH1 prevents ferroptosis by acting as an antioxidant through BH4/BH2. High-expressed BH4 cells can prevent oxidative damage by producing CoQ10, which prevents oxidation directly and prevents cellular ferroptosis (25).

### 2.5 Iron overload

The transferrin receptor (TFRC) recognizes Fe (3+) bound to transferrin (TF) in the serum and facilitates its entry into the cell. Once inside, Fe (3+) is reduced to Fe (2+) and stored in the iron pool. An excess of Fe (2+) activates iron-containing enzymes, which react with H2O2 to generate a significant amount of reactive oxygen species (ROS), leading to ferroptosis (26). When the stored ferritin is recognized by NCOA4, it is recruited into the autophage, causing oxidative damage, a process also known as ferritinophagy, which can help induce ferroptosis (5). Simultaneously, the susceptibility to ferroptosis is increased when ferritinophagy takes place, which results in a significant amount of free iron (27).

## 3 Regulation of ferroptosis in ovarian cancer

### 3.1 Signal pathway

#### 3.1.1 HIPPO pathway

The hippo pathway is a tumor-suppressing pathway that can detect and control cell density. The two main transcript coactivators of this pathway are Yes-associated protein 1 (YAP) and transcription coactivator with PDZ-binding motif (TAZ). Transcription enhancement-related domains (TEAD) members can interact with TAP to mediate multi-cancer proliferation, renewal, transfer, and drug resistance (28). The Hippo pathway controls ferroptosis in ovarian cancer through YAP and TAZ. When TAZ is overexpressed, OVCA cells become susceptible to ferroptosis. TAZ also causes ferroptosis by controlling the level of its direct target gene, angiooietin-like 4(ANGPTL4), which in turn controls the activity of NADPH oxidase 2 (NOX2) (29). When TAZ is overexpressed, OVCA cells become susceptible to ferroptosis. When SKP2 or YAP are removed, lipid oxidation is inhibited during the ferroptosis inducer (30).Therefore, the Hippo pathway may be a major target in ovarian tumor cell ferroptosis and play a crucial role in controlling the susceptibility of ferroptosis inducers to ovarian tumor ferroptosis.

#### 3.1.2 Nrf2 pathway

To keep cell metabolism, oxidation restoration, protein sedimentation balance, and antioxidation going, nuclear factor erythroid 2-related factor 2 (NRf2) is a transcription factor that is very important. It has been discovered that numerous significant ferroptosis regulators are NRF2’s target genes (31). For example, research has discovered that NRF2 can govern the ferroptosis of tumor cells through many downstream targets such as GPX4 channels (32, 33), HMOX1 (34), ferritinophagy activating proteins (ATG5 etc.) (31), while NRF 2 has also been reported to be connected with the prognosis of tumors (35).

Several studies have found that NRF2 is a regulatory factor in ovarian tumor ferroptosis. Apatinib can induce ferroptosis by combining olaparib with NRf2, inhibiting GPX4 expression (36). Pachymaran can induce ferroptosis by lowering NRF2 mRNA to raise Fe2+ and lower levels of NRF2,HO-1 and GPX4 proteins in ovarian cancer cells (37). Norcantharidin (NCTD) can also regulate ferroptosis by acting on the NRF2/HO-1/GPX4/xCT axis, leading to ferroptosis of ovarian cancer cells by inhibiting NRF2 (38). Tripterygium glycosides, by targeting the NRF2/GPX4 signal axis, disrupt the stability of the oxidation restoration reaction and induce ferroptosis in ovarian tumor cells, thereby enhancing the chemical susceptibility of ovarian cancer to cisplatin (39). Wang et al. found that eryodictyol reduced NRF2 expression in mouse tumor tissue and regulated ferroptosis in ovarian cancer through NRF2/HO-1/NQO1 signal pathways (40). Furthermore, NRF2 can enhance erastin-induced ferroptosis resistance by upgrading the expression of CBS in anti-erastin cells in the transsulfuration pathway (41). Chelerythrine (CHE), widely recognized as an anticancer agent, was found by Jia et al. to exert inhibitory effects on ovarian cancer cell growth. This inhibition was achieved through its action on Nrf2, which mediated the expression of ferroptosis-related proteins and subsequently promoted ferroptosis (42). NRF2 can also play a role in ferritin synthesis and degradation NRF2 regulates ferritin by HECT and RLD domains containing E3 ubiquitin protein ligase 2(HERC2), NCOA4 and vesicle-associated membrane protein 8(VAMP8). In addition, ovarian cancer cells with high NFE2L2/NRF2 expression have been found to increase sensitivity to the ferroptosis inducer (43).This demonstrates that NRF2 is an important treatment target for ovarian cancer and plays a role in a variety of regulatory pathways. While other ferroptosis regulating factors in ovarian cancer that are regulated by NRF2 are not yet fully studied, future studies of NRf2 in ovary cancer are to be expected.

#### 3.1.3 P53 pathway

P53 is a tumor suppressor protein that plays a powerful role in cell aging, death, and DNA damage repair. In recent years, more and more research has found that P53 plays a crucial role in ferroptosis. The regulatory effect of P53 is two-way and has a different effect as the environment changes. When lipid peroxide is slight, it inhibits the occurrence of ferroptosis, helping the cell to survive. However, when lipid peroxide is persistent and severe, it induces ferroptosis, helping tumor cell death (44). For the GPX4-Xc system, P53 can combine and degrade SLC7A11, enhance the expression of SAT1 and GLS2 in cells (5, 13), and reduce the production of GSH by inhibiting CBS (45) expression, thereby suppressing GPX4.When p53 elevates SAT1 expression, it can indirectly promote ALOX15 elevation, promoting lipid peroxide (46).Studies have also shown that p53 can inhibit the development of ferroptosis by blocking dipeptidyl-peptidase-4 (DPP4) activity, limiting the peroxidation of lipids (47). P53 inhibits TFR1 and ZRT/IRT-like Protein 14 (ZIP14),reducing cellular Fe (2+) (48). p53 can also inhibit the occurrence of ferroptosis by reactivating the two-minute binoculars (MDM2) in mice, activating FSP1 (49).

Through p53, PARP inhibitors block SLC7A11 expression in ovarian cancer, which lowers GSH synthesis and increases lipid peroxide and ferroptosis (50). Apatinib combined with olaparib causes ferroptosis in ovarian cancer through p53 dependent way (36). A lack of MEX3A causes the p53 protein to become more stable, which prevents ferroptosis and encourages ovarian cancer (51). To encourage OVCAR-3-cell ferroptosis, ursolic acid (UA) can trigger the JNK/p53 signal pathway (52).

### 3.2 Gene

#### 3.2.1 SCD1

Stearoyl-CoA Desaturase 1(SCD1) is an enzyme that catalyzes the synthesis of monounsaturated fatty acids in ovarian cancer cells and is highly expressed in ovary tumor cells (53). Inhibition or absence of the SCD1 gene can induce cellular ferroptosis. SCD1/FADS2 has a positive ratio to the level of unsaturated fatty acids, which can regulate lipid peroxide. Inhibiting SCD1/FADS2 can also directly degrade GPX4, thereby inducing the development of ferroptosis (54). Menin-mixed-lineage leukemia(Menin-MLL) inhibitor MI-463 can mediate ferroptosis in cancer cells through lipid peroxide regulated by SCD1 (55). Furthermore, in ovarian cancer cells, agrimonolide can target SCD1 as a new ferroptosis inducer (56). The ferroptosis pathway is one of the SCD1 routes that TP53 (13) can regulate, indicating that ovarian cancer with TP53 mutations may respond better to a SCD1 inhibitor. In addition to increasing ovarian sensitivity to ferroptosis, SCD1 medication suppression may be more advantageous for TP53-mutated ovarian malignancies (57). TESFAY found that ferroptosis inhibitor erastin can be used in conjunction with SCD1-inhibitors A939572 to regulate lipid metabolism, significantly enhancing the anti-tumor effect of the induced ferroptosis inducer in the ovarian cells, increasing ovarian cell susceptibility to ferroptosis inducers (53). Treatment with the SCD1-inhibitors MF-438, CAY10566, and 939572 makes ovarian carcinoma cells more susceptible to the death ferroptosis-inducers RSL3 and Erastin (58). Treatment of ovarian tumor cells with SCD1/FADS2 inhibitors in combination with ferroptosislatin can raise the rate of apoptosis, decrease the rate of cell mobility and tumor metastasis, and increase the sensitivity of ovarian tumor cells to ferroptosislatin (54). According to a number of studies, SCD1 inhibitors in ovarian cancer can dramatically increase the effect of ferroptosis and aid in tumor cell regression, in addition to increasing the sensitivity of ferroptosis inducers. This suggests that SCD1 may one day serve as a novel therapeutic.

#### 3.2.2 FZD7

Wnt receptor Frizzled-7 (FZD7) is a transmembrane receptor that functions beyond the signals of both the canonical pathway and the Wnt/Ca2+ pathway. Overexpression of FZD7 can drive the development of ovarian tumors via the Wnt signal pathway (59). FZD7 directly links to the expression of GSS, GSR, GPX2, and IDH genes related to GSH metabolism. In ovarian cancer tissue, FZD7 can activate the carcinogen P63, enhance the expression of GPX4, prevent tumor cell ferroptosis, and decrease the susceptibility of drug-resistant ovarian cancer cells to ferroptosis (60). In addition, miR-1-3p significantly improves the sensitivity of ovarian cancer cells to Erastin or RSL3-induced ferroptosis by decreasing FZD7 expression (61). This shows that treating ovarian cancer cells resistant to platinum may benefit from targeting FZD7. Simultaneously, an innovative prospect of combining a ferroptosis inducer with a FZD7 inhibitor emerged.

#### 3.2.3 SNAI2

Research has demonstrated strong expression of SNAI2 in ovarian cancer cells, directly linking this expression to the cells’ ability to survive, proliferate, invade, and spread. The promoter area of SLC7A11 is bound by SNAI2, and when SNAI2 is blocked, SLC7A11 expression is down-regulated, which causes ferroptosis in ovarian carcinoma cells (62). A leucine zipper transcription factor called CCAAT/enhancer binding protein gamma(CEBPG) has also been found to enhance transcription in SLC7A11 and promote GPX4 expression, thereby inhibiting ferroptosis in the OC. and leading to tumor cell development. Additionally, at the clinical level, CEBPG expression has been observed to be associated with an adverse prognosis in patients with OC (63). The E3 ubiquitin ligase 3-hydroxy-3-methylglutaryl reductase degradation (HRD1) inhibits the growth of tumors in a variety of cancer forms. HRD1 has the capacity to control ubiquitination and OC cell stability. Through increasing SLC7A11 degradation, HRD1 interacts with SLC7A11 in OC cells to encourage ferroptosis and prevent the growth of OC tumors (64).

#### 3.2.4 PARP

Poly (ADP-ribose) polymerases (PARP) are involved in cellular processes such as DNA repair, transcription, metabolic regulation and cell death (65). PARP inhibitors are currently an effective treatment for BRCA mutant ovarian cancer (66). PARPi reduces the expression of SLC7A11 in a p53 dependent way, thereby reducing the biosynthesis of GSH and promoting lipid peroxide and ferroptosis. It was also found that PARPi was associated with the expression of CBS, FSP-1,etc. (50).Olaparib is a classic PARP inhibitor for treating BRCA mutant ovarian cancer. The combined treatment of olaparib and arsenic trioxide (ATO) activates the AMPKα pathway and inhibits SCD1 expression, resulting in a significant increase in lipid peroxide in ovarian cancer cells, which induces the occurrence of ferroptosis (67).However, BRCA (wild type) ovarian cancer can produce olaparib resistance by repairing PARPi-induced DNA damage. On this basis, PARPi combined with FINS targeting SLC7A11 or GPX4 can synergistically enhance ferroptosis, thereby producing an effective tumor suppression effect against BRCA (wild type) ovarian cancer (50).Similarly, Apatinib combined with olaparib reduced GPX4 by inhibiting expression of NRF2 and autophagy, inducing ferroptosis in ovarian cancer. However, in P53 (wild type) ovarian cancer cells, the p53 activator RITA can increase the sensitivity of resistant cells to ferroptosis, enhancing the effect of ferroptosis (36).PARP inhibitors, in addition to their therapeutic effects on BRCA mutant ovarian cancer cells, have also been combined with ferroptosis inducers or critical factors to enhance the sensitivity of mutant ovary cancer to ferroptosis.

#### 3.2.5 ARDHEP 15

In the clinical treatment of malignancies, microtubule-targeted agents (MTA) are useful chemotherapeutic agents, and the interaction of microtubulin with VDAC provides a novel target for inducing ferroptosis in cancer cells (68). Tubulin polymerization can be inhibited by the newly synthesized novel aroyl diheterocyclic pyrrole (ARDHEP) 15. ARDHEP may induce ferroptosis in ovarian tumor cells and prevent tumor cell growth by upregulating GPX4, increasing intracellular ROS and Fe(2+) buildup, and stimulating cellular oxidative damage (69). It is a novel therapeutic target for tumors.

ALDH3A2 is a member of the ALDH family. Studies have shown that removing ALDH3A2 boosts lipid metabolism and, when combined with GPX4, helps prevent ferroptosis. Additionally, the expression of ALDH3A2 is directly correlated with the ferroptosis susceptibility of ovarian cancer cells, which can prevent ferroptosis in ovarian cancerous cells (69).

#### 3.2.6 RNA

Microscopic RNAs (miRNAs) are a class of endogenous expressions of non-coding RNA that are highly significant in biological processes such as differentiation, proliferation, mortality, etc. Recent research has also discovered that miRNAs regulate ferroptosis, connecting them to numerous diseases, including cancer (70). By directly combining the 3’ Untranslated Regions (UTR) with ACSL4, mir-424-5p inhibits the expression of ACSL4, thereby reducing the ferroptosis induced by erastin and RSL3, thereby lowering the sensitivity of ovarian cancer tissue to ferroptosis, ultimately leading to the malignant progression of OC (71). Researchers found that Extrinsic Fe and DFO target miR-485-3P and miR-194 to regulate the expression of FPN. Large-scale induction of miR-485-3P expression can reduce intraocular FPN, which raises Fe2+ in ovarian cancer cells and causes the cells to undergo ferroptosis (72). By boosting miR-382-5p to lower SLC7A11 and so preventing the proliferation, invasion, and transfer of ovarian cancer cells, lidocaine also causes a buildup of iron content and reactive oxygen species (ROS) in the OC. cells (73). lncRNA is a non-coding RNA with a length longer than 200 nucleocarbons and plays a significant role in epigenetic regulation, cell cycle regulation, and cell differentiation regulation (67).lncRNA ADAMTS9-AS1 can block the process of ferroptosis in OC cells via modulating the mir-587/SLC7A11 axis, which can further the malignant growth of OC cells (73). JIN et al. found that LncRNA CACNA1G-AS1 can stimulate the growth and transfer of ovarian cancer cells through the FTH1-IGF2BP1 axis to regulate the expression of FTH1 and inhibit ferritinophagy (74). According to recent research, circRNA regulates ferroptosis, anemia, metabolism, tumor growth, and anemia (75). CircRNASnx12 improves immunomodulatory resistance in ovarian cancer by targeting miR-194-5p/SLC7A11 pathways to block ferroptosis. CircRNASnx12 can therefore serve as an effective therapeutic target for overcoming cisplatin resistance (76) (**Table 1**).

**Table 1:** Genes involved in ferroptosis of ovarian cancer cell.

Gene	Mechanism	Function	Reference
TAZ	Target ANGPTL4/NOX2	induce ferroptosis and increase sensitivity	(29)
YAP	Target SKP2	induce ferroptosis and increase sensitivity	(30)
NRF2	Target GPX4/HO-1/HMOX1/CBS	inhibit ferroptosis	(41)
P53	GPX4/Xc system	induce ferroptosis	(77)
PARP	GPX4/Xc system/P53/FSP-1	induce ferroptosis and increase sensitivity	(50)
MEX3A	Target P53	induce ferroptosis	(51)
SCD1	Target GPX4	inhibit ferroptosis	(54, 57)
F7D7	Target Wnt/GPX4/TP63	inhibit ferroptosis and decrease sensitivity	(60)
miR-1-3p	Target F7D7	induce ferroptosis and increase sensitivity	(61)
SNAI2	Target SLC7A11	inhibit ferroptosis	(62)
GEBPG	Target SLC7A11	inhibit ferroptosis	(63)
HRD1	Target SLC7A11	induce ferroptosis	(64)
ALDH3A2	Target GPX4	inhibit ferroptosis and decrease sensitivity	(78)
mir-424-5p	Target ACSL4	inhibit ferroptosis and decrease sensitivity	(71)
miR-485-3P	Target FPN	induce ferroptosis	(72)
miR-194	Target FPN	induce ferroptosis	(76)
miR-382-5p	Target SLC7A11	induce ferroptosis	(73)
lncRNA ADAMTS9-AS1	mir-587/SLC7A11	inhibit ferroptosis	(79)
lncRNA CACNA1G-AS1	Target FTH1-IGF2BP1	inhibit ferroptosis	(74)
circRNASnx12	Target miR-194-5p/SLC7A11	inhibit ferroptosis and increase sensitivity	(76)
HNF1	Target P53	inhibit ferroptosis and increase sensitivity	(80, 81)
FDX1	Target ROS	inhint ferroptosis and decrease sensitivity	(82)
c-myc	Target NCOA4/HMGB1	inhibit ferroptosis	(83)
MTHFR	Target HMOX1	inhibit ferroptosis	(84)

### 3.3 Drug therapy

Researchers are still studying the development of ferroptosis-related drugs for ovarian cancer despite the increasing clarity on many mechanisms of ferroptosis. The thioredoxin reductase auranofin, is used in conjunction with the MENIN-MLL inhibitor MI-463 to induce ferroptosis in ovarian tumor cells (55). Artesunate (ART) is a widely used anti-malaria drug that has been studied to find multiple cellular responses involved in tumor cells, such as mortality, malnutrition, ferroptosis, etc. (85). Greenshields and others found that ART treatment induces ovarian cancer cells to produce a large amount of ROS, which exerts a potent anti-proliferative and cell-toxic effect on ovarian cancers. At the same time, neither the use of mortality inhibitors nor ferroptosis related-inhibitors can completely eliminate the effect of ART, indicating that ART has a combined inhibitory effect on ovarian cancer tumor cells in a variety of ways (86).Octreotide, an FDA-approved medication that is commonly used in the clinical treatment of ovarian cancer, can directly decrease the expression of GPX4 inducing ferroptosis (87). A rating system of ferroptosis-related genes constructed using TCGA mRNA expression data found Dimethyloxalylglycine (DMOG) to be a potentially sensitive drug for ovarian cancer (88). Large doses of selenium induce cell death mediated by ferroptosis through abnormal GPx4 and lipid peroxide mechanisms, thereby producing anti-cancer effects. High doses of selenium have been speculated to lead to GPx4 deficiency through the Wnt/β-catenin signal pathway (89). After the treatment of ovarian cancer stem cells (OCSCs) with anisomycin, the levels of triphosadenine and total glutathione were found to be significantly reduced, Fe2+ levels increased, lipid peroxide increased, and the activity of OCSCs significantly decreased. Furthermore, anisomycin reduces the level of transcription of gene clusters that encode pathways related to the regulation of ferroptosis, such as glutathione metabolism and the autophagy signal transduction pathway. The genes of the core factor ATF4 of these two pathways are significantly expressed in ovarian cancer tissue and are associated with a poor prognosis. Thus, anisomycin may induce ferroptosis in ovarian cancer stem cells by reducing ATF4 to regulate glutathione metabolism (90). Leukemia inhibitory factor (LIF) and its receptor (LIFR) can induce ferroptosis via the GPX4 system. Additionally, LIF and LIFR have been found to act on M1 macrophages, enhancing the activity of CD8+ T cells and thereby regulating the immunogenicity of ovarian cancer cells. However, the specific mechanisms and pathways involved in this process remain unclear (91).

As a new direction in ferroptosis applications, nanomaterials are safer, more durable and more accurate in applications that induce ferroptosis to produce ovarian tumor suppression (92). In addition, nanomaterials have been found to enhance the immunotherapy effect of induced ferroptosis (93). Superparamagnetic iron oxid spio-serum can effectively induce lipid peroxide and produce a large amount of toxic ROS by reducing the expression of the ferroptosis related proteins SLC7A11 and GPX4 in OVCA cells, play synergies with p53 and promote the occurrence of ferroptosis in ovarian cancer cells (77). Biomimetic magnetic nanoparticles Fe3O4-SAS @ PLT are constructed by Fe3O4 and a platelet membrane covering containing sulfasalazine (SAS), which can increase the sensitivity to ferroptosis, inducing ferroptosis by inhibiting the Xc-system. In addition, Fe3O4-SAS @ PLT can also produce mild immunogenicity that triggers the immunotherapy response to ovarian cancer (94). Chemokinetic therapy (CDT) is considered one of the most promising cancer treatments, mainly through the Fenton reaction. As a Fenton reagent, iron nitroprusside, (FeNP) has a therapeutic effect on ovarian cancer organs originating from high-grade serous ovarian carcinoma (HGSOC) by inhibiting GPX4’s involvement in ferroptosis (95) (**Table 2**).

**Table 2:** The drug that regulate ferroptosis and sensitivity of ovarian tumor cell to chemotherapy.

DRUG	Mechanism	Function	Reference
erastin	target ROS	induce ferroptosis	(96)
Apatinib	target NRF2/GPX4/p53	inhibit ferroptosis	(36)
pachymaran	target NRF2/HO-1	inhibit ferroptosis	(37)
NCTD	target NRF2/GPX4	induce ferroptosis	(38)
tripterygium glycosides	target NRF2/HO-1/NQO1	induce ferroptosis and increase sensitivity	(39)
eriodictyol	target Nrf2/HO-1/NQO1	induce ferroptosis	(40)
UA	target JNK/P53	induce ferroptosis	(52)
MI-463	target SCD1	induce ferroptosis	(54)
Agrimonolide	target SCD1	induce ferroptosis	(56)
ARDHEP15	target GPX4	induce ferroptosis	(69)
ART	target mTOR/ROS	induce ferroptosis	(86)
Octreotide	target GPX4	induce ferroptosis	(87)
Sodium Selenite	target Wnt/β-catenin/GPX4	induce ferroptosis	(97)
Anisomycin	target ATF4/GPX4	induce ferroptosis	(90)
spio=serum	target SLC7A11/GPX4	induce ferroptosis	(77)
Fe3O4 - SAS @ PL	target Xc system	induce ferroptosis	(98)
FeNP	target GPX4	induce ferroptosis	(95)
ferlixit	target Fe2+	induce ferroptosis	(99)
Sorafenib	target SLC7A11/GPX8	induce ferroptosis	(100)
MAP30	target Ca2+	induce ferroptosis and increase sensitivity	(101)
oncolytic vaccinia virus	target CXCR4	induce ferroptosis and increase sensitivity	(102)
GALNT14	EGFR/mTOR	induce ferroptosis and increase sensitivity	(103)
Chelerythrine	target Nrf2	induce ferroptosis	(42)
LIF	Target GPX4	induce ferroptosis	(91)

Although research on medications linked to ferroptosis is still ongoing, there is still hope for significant future developments in ovarian cancer management.

### 3.4 Enhancement of chemoresistance

Currently, the main clinical treatments for ovarian cancer are surgery and chemotherapy with paclitaxel combined with platinum drugs. However, the prognosis for ovarian cancer patients who are susceptible to chemotherapy resistance is poor. Nevertheless, research has shown that ferroptosis inducers can improve the chemotherapy sensitivity of ovarian cancer cells (104). The ferroptosis inducer erastin has been shown to activate the apoptosis pathway, which may increase the sensitivity of HEY and SKOV3 cells to cisplatin (105). Furthermore, expression due to ATP binding cassette subfamily B member 1 (ABCB1) in OVCA cells that are resistant to another chemotherapeutic drug, dositase, erastin exhibits a strong reversal effect of ABCB1, increasing the susceptibility of OVCAC cells to docetaxel (94).

The sensitivity of cells to ferroptosis and ferroptosis conditions is strongly correlated, and the iron compound ferlixit joint erastin can overcome the chemotherapeutic resistance of ferroptosis ovarian cancer (99). Studies have also demonstrated that the acquired synthesis of cystine and glutathione impacts carboplatin resistance in ovarian cancer. Hepatocyte nuclear factor-1-beta (HNF1) can promote glutathione synthesis to avoid carboplatin resistance to ovarian clear cell carcinoma (80). Simultaneously, P53 was identified as a key pathway in the bioinformatic analysis of the resistance of HNF1 to ovarian cancer (81). The loss of Fdx1 mediated by siRNA in cisplatin-resistant cells is potentiated by an elevation in mitochondrial membrane potential and cisplatin-induced lipid peroxidation, ultimately leading to ferroptosis. Immunohistochemical analysis of clinical specimens from ovarian cancer patients revealed higher expression levels of Fdx1 in cisplatin-resistant specimens compared to cisplatin-sensitive ones. Fdx1 may be a new and appropriate diagnostic and prognostic marker and therapeutic molecular target for the treatment of COVID-19 (82). SLC7A11 and GPX4 high expression levels linked to platinum resistance in EOC patients. The combined expression of SLC7A11 and GPX4 may be a significant independent prognostic factor and a potential treatment target for EOC patients (106). For example, Tripterygium glycosides target the NRF2/GPX4 signal axis and mess up the stable reaction of oxidation restoration. They also cause ferroptosis in A2780/DDP cells and make ovarian cancer more likely to respond to cisplatin (39).

Statistics for progression-free survival and clinically significant improvement were found in patients with ovary cancer who were treated with sorafenib combined with topotecan maintenance therapy (100). MAP30 protein from Momordica charantia and cisplatin can synergistically induce ferroptosis in ovarian cancer cells by altering metabolism (101). GALNT14, a member of the acetylgalactosaminyltransferases family, which can regulate the stability of EGFR proteins to inhibit the EGFR/mTOR pathway, has significantly higher levels of GALNT14 in cisplatin resistance ovarian cancer tissue compared to cisplatin sensitive ovarian cancer tissue. The combination of cisplatin and the mTOR inhibitor GALNT14 had a cumulative effect by promoting apoptosis and ferroptosis of ovarian cancer cells, which may offer a new target for overcoming cisplatin resistance in ovarian cancer (103).By increasing ROS, lipid peroxidation, and iron homeostasis in high-OXPHOS high-grade serous ovarian cancer (HGSOC), the promyelocytic leukemia protein-peroxisome proliferator-activated receptor gamma coactivator-1a (PML-PGC-1a) axis can help make ovarian cancer more sensitive to chemotherapy. These features are regulatory mechanisms for ferroptosis. It is therefore suspected that this mechanism may improve resistance in ovarian cancer by modulating ferroptosis (107). Furthermore, it was discovered in Zhang ‘s study that chemotherapeutic drugs can also result in lipid peroxidation through an excess of ROS, which can lead to ferroptosis in normal ovarian cells (108). Figuring out the exact way ferroptosis works and what it targets in chemotherapy could help make ovarian cancer cells more sensitive to chemotherapy drugs while keeping healthy ovarian tissue as safe as possible.

## 4 Immunotherapy

The study of immunotherapy in the field of ovarian cancer is a hot topic right now. The three main approaches are tumor antigen vaccines, monoclonal antibodies that target the expression of immune checkpoint inhibitors, and immunostimulatory cytokines. Cancer cells can escape immune therapy by modulating immune checkpoint pathways. The study found that when TYRO3 inhibits ferroptosis by regulating ferroptosis pathways such as NRF2, Xc system, tumor cells express high resistance to anti-PD-1/PD-L1 therapy (109).In addition, IFN γ released by CD8(+)T cells has been found to promote ferroptosis by acting on SLC3A2 and SLC7A11 on cancer cells, which also enhances anti-tumor immunotherapy against PD-L1 (110). Studies have shown that ovarian cancer is immunogenic (111), and that immunotherapy can extend the survival period of patients with ovarian cancer while lowering the recurrence rate (111). This means that using ferroptosis inducers along with immune checkpoint inhibitors might work well to treat ovarian tumors.

### 4.1 DAMPs

**Figure 2:**
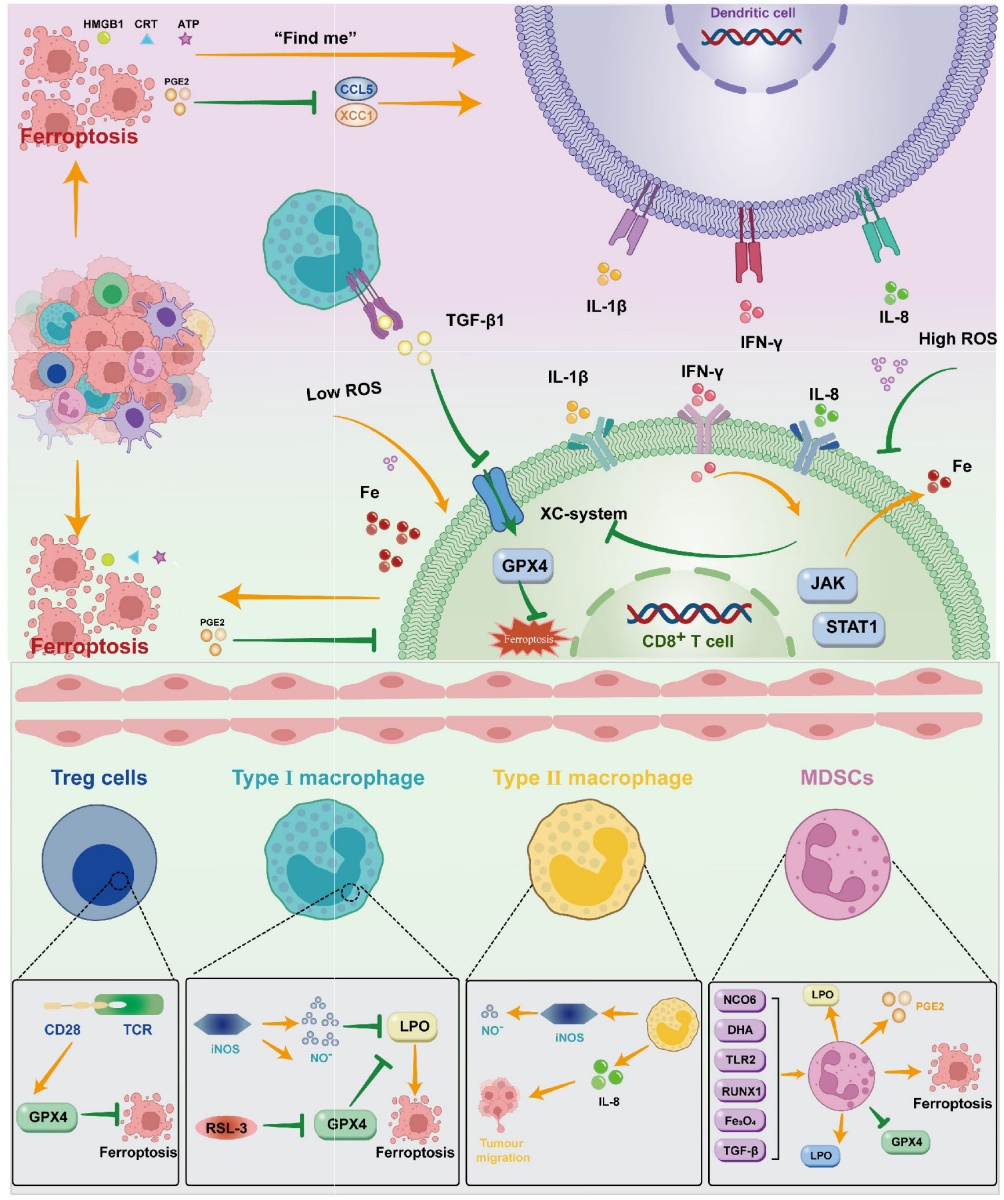
DAMPs such as HMGB1, CRT, ATP, and PGE2 are created when ferroptosis occurs in ovarian cancer cells. These molecules primarily operate on DC through various signals; DC can also act on CD8+T cells by releasing cytokines like IL-1β, IFN-γ, IL-8, and so on. T cells can control the incidence of ferroptosis by inhibiting the Xc system through JAK/STAT1 and promoting Fe through the release of IFN-γ. Macrophages mainly influence the Xc system by means of TGF-β1. To control ferroptosis, immunosuppressive cells can operate on the GPX4 system in a variety of ways.

When tumor cells die due to external stimuli, the process from the non-immunogenic to the immunogenic immune response of the mediated organism is known as immune cell death (ICD). Tumor cells experiencing ICD can release large amounts of cell content into the extracellular space through sudden and uncontrolled cell death. The damage signal molecule is called the damage-associated molecular pattern (DAMPs). The presence of DAMPs in extracellular space triggers a strong immune response, drawing in more phagocytes and other immune cells to eliminate the threat and encourage tissue repair (112). Some anti-tumor drugs can induce ICD through DAMPs (112), recruit immune-inflammatory cells, release a large amount of inflammatory agents, and cause inflammation responses that cause the destruction of normal surrounding tissue, stimulate the formation of neonatal blood vessels, increase vascular permeability, weaken adaptive immune responses, promote tumor development, cancer transfer and tumor resistance (113). Ferroptosis kills cancer cells by enhancing immune cell activity (114). The DAMPs that primarily contribute to ferroptosis include HMGB1, CRT, ATP, and others. The release of ATP from dead cells is a self-dependent process. The presence of ATP serves as a “find me” signal in extracellular space, which is a chemoattractant of the DC precursor. ATP binds to the P2RX7 receptor on DCs, facilitating inflammasome mediated secretion of interleukin 1β (IL-1β), and the binding signals stimulate the production of the pro-inflammatory cytokine IL8 (115), and increase the recruitment of neutrophils and phagocytic potential.CRT is also a recognized DAMP molecule that plays a key role in the onset of immune cell death. The CRT on the membrane of the dead cell serves as a “eat me” signal to the APCs and triggers immune stimulation. HMGB1 is currently mainly believed to play a role through the promotion of inflammatory mediators, achieving inflammation responses, and initiating immunotherapy in ferroptosis (116) (**Figure 2**).

### 4.2 Immune cells

Immunol cells can perform anti-tumor immune functions by releasing cytokines that promote the ferroptosis activity of tumor cells. CTL-released IFNγ activates the Janus kinase (JAK) signal and signal transducer and activator of transcription 1 (STAT1) pathways, thereby reducing the expression of SLC3A2 and SLC7A11 to inhibit Xc system expression, increasing the iron content stored in the cell, and thus inducing ferroptosis (117). IFNγ released by CD8(+)T cells inhibits the expression of SLC7A11 synergistically, leading to the activation of ferroptosis, thereby enhancing anti-PD-L1 anti-tumor immunotherapy, enhancing lipid oxidation and ferroptosis in tumors, and improving tumor control (110). Similarly, the transformed growth factor-β (TGF-β1) released by macrophageal cells can inhibit transcription of the Xc system through SMAD signals, thereby promoting ferroptosis (118). The SAPE-OOH on the surface of the deferrous cell, as an eat-me signal, guides swallowing by targeting TLR 2 on the macrophageal cell (119) (**Figure 2**).

### 4.3 Immunosuppressive cells

Some immunosuppressive cells can antagonize ferroptosis through high expression of GPX4 or other components (110). In addition, Gpx4 can suppress anti-tumor immunity by promoting Treg cell survival in the tumor (120). In Tregs with TCR/CD28 co-stimulation activation,GPX4 expression was promoted, thereby reducing the occurrence of ferroptosis (121).With the absence of GPX4,M1 cells express high amounts of nitric oxide synthase (iNOS) and create more NO free radicals (NO-), which can have an impact akin to GPX4. (NO-) down-regulates the expression of ferroptosis (122). Intratumoral prostaglandin E2 (PGE2) is an immunosuppressive mediator that directly inhibits cytotoxic T cell activity (123). It also decreases the number of DCs infiltrating into the TME by inhibiting the chemokines CCL5 and XCL1 (124). The release of PGE2 is linked to the induction of ferroptosis in tumor cell (8, 125, 126). PGE2 overproduction inhibits the tumor immune response and promotes tumor immune evasion (127).

Immunocytosuppressive tumors, or “cold tumors,” do not have tumor-infiltrating T cells, which do not respond to immune checkpoint inhibitors. In cold tumors, T cells lack cystathionase and Xc transporters, while myeloid-derived suppressor cells (MDSCs) lack ASC neutral amino acid transporters and limiting extracellular cysteine, inhibiting T cell activation (128), and its TME is immersed in various immunosuppressive cells (129, 130). We are aware that MDSC is an immunosuppressive cell in ovarian tumor cells. Several studies have demonstrated that MDSC has an immunosuppressive impact by preventing CD8+T cells from infiltrating TME (131, 132), increasing PGE2 to induce PD-L1 expression in ovarian tumor cells (133). As the research goes deeper, there are greater expectations for the combination of ICIs with ferroptosis inducers. Immunosuppressant M2 can be re-polarized to M1 (98, 134) when ferroptosis is caused by certain circumstances, providing an environment for ICIs. Research has demonstrated that CD36 facilitates the absorption of fatty acids by CD8 T cells in TME that have invaded tumors, inducing lipid peroxide and ferroptosis, resulting in reduced cytotoxic cytokine generation and impaired anti-tumor capacity. Blocking CD36 or inhibiting ferroptosis in CD8 T cells effectively restores its anti-tumor activity. More importantly, it has a greater antitumor effect when combined with anti-PD-1 antibodies (135). As an inducer of ferroptosis, erastin has little effect on autoimmune activity. However, when combined with oncolytic vaccinia virus (OVV), erastin promotes therapeutic effectiveness and anti-tumor immunity by increasing the number of activated DCs and promoting the activity of tumor-specific CD8+T cells in the tumor (102). The combination also led to increased expression of PD-1 and CTLA-4 in the TME. It provides a molecular basis for future ICIS combination therapy. Therefore, the combination of ferroptosis inducers with ICIs enhances tumor immunotherapy.

However, the combination of ferroptosis inducers with ICIs is also likely to increase immunosuppression, which is mainly the role played by MDSC. Ferroptosis of PMN MDSCs in TME gives them stronger immunosuppressive capabilities that are sufficient to convert non-inhibiting PMNs into immune-inhibitory PMN-MDSCs. Even though ferroptosis lowers the number of PMN MDSCs in the TME, it also increases the release of molecules that weaken the immune system, which stops T cells from working. In addition, PMN MDSCs in TME are known to produce PGE2, which undermines the anti-tumor function of innate and adaptive immune cells, inhibits ferroptosis, protects PMN MDSC, and blocks the release of immunosuppressive factors, thereby facilitating the conversion of PMN MDSC to classical non-inhibitory PMN. In some models, the immunosuppressive effects of ferroptosis in PMN-MDSC can exceed its tumor-suppressive effect on cancer cell death. PMN-MDSCs can also rely on peroxide enzymes for lipid peroxidation and transfer lipid to DC cells, blocking the cross-presentation of DC, thus exerting pro-tumor activity (136). The study found that GPx4 deficiency did not inhibit the development of tumors, instead, the GPx4-related ferroptosis caused the cytotoxic CD8+T cell CXCL10 dependent immersion, which was offset by the PD-L1 upgradation of the tumor cell and the significant HMGB1-mediated MDSC infiltration, resulting in a tumor-inhibiting immune response. Therefore, the combination of ferroptosis inducers with ICIs has not yet been clearly concluded. However, there are also ferroptosis inducers that can inhibit MDSC. Dihydroartemisinin (DHA) has a marked anti-tumor and inducing ferroptosis function in tumors, which can be achieved using the PDAC orthotopic tumor model, which significantly inhibits MDSC (137). TLR2 and Runx1 can also regulate MDSC through the ferroptosis path (124). The combination of induced ferroptosis with PD-L1 and MDSC blockage induced ferroptosis doesn’t rely on the presence of particular mutations in tumor cells, meaning that any type of tumor may be treated with this combination therapy. The TME reaction to ferroptosis will determine how well the therapy works (138). Zhu et al. found that Asah2 is highly expressed in tumor-infiltrating MDSCs, which can regulate the ferroptosis of MDSCs through the P53-HMOX1 pathway. The use of the Asah2 inhibitor NC06 to target ASAH2 to induce MDSC ferroptosis is a potentially effective therapy for inhibiting the MDSC accumulation in cancer immunotherapy (139). In addition, Zhang and others constructed a biomimetic magnetosome composed of Fe3O4 magnetic nanocluster (NC) as a core, loaded with TGF-β inhibitors and PD-1 antibodies (Pa).When entering the tumor, Pa and Ti synergistically form an immunogenic micro-environment that increases the amount of H2O2 in the polarized M1 macrophage cells, thereby promoting the Fenton reaction released with NCs. Meanwhile, the produced (OH) induces the ferroptosis of ovarian tumor cells, and the micro-environmental immunogenicity is increased by the exposed tumor antigen. The synergistic effect of ferroptosis and immune therapy in ovarian cancer was generated (140). This gives us the clue that it may be possible to use the ferroptosis inducer in conjunction with ICIs for immunotherapy of tumors in cases where MDSC is suppressed.

It also gives us new ideas for ovarian cancer immunotherapy. The study discovered that ferroptosis was closely associated with ovarian tumor immunity (141). However, in contrast to other tumors, current studies on ferroptosis in immunotherapy are more likely to induce ferroptosis release of DAMP, causing tumor development and a poor prognosis. For instance, C-MYC inhibits ferroptosis induced HMGB1 release mediated by NCOA4 in ovarian cancer cells (83). Low-concentration erastin by STAT3 mediated M2 polarization of macrophage cells increased ferroptosis resistance to ovarian cancer. The macrophage cells processed by erastin also secrete the key cytokines IL-8, which encourage the invasion and migration of anti-ferroptosis O.C. cells (96). However, studies have also found that immune cells can have an anti-tumor effect by inducing the ferroptosis of ovarian cells. ICIs can induce CD8+ T cells to trigger ferroptosis in mouse ovarian tumor cells (142). Immunotherapy-activated CD8+T cells can release IFN-γ and enhance the anti-tumor effect of immunotherapy by inducing ovarian cancer cell ferroptosis. IFN-γ kills mouse OVCA cells ID8 through inhibiting the Xc system, increasing lipid ROS, and reducing tumor growth (117). The study found that inhibition of ovarian cancer metastasis was achieved through targeted MDSC immersion TME (143). By encouraging the release of C5 by ovarian cancer cells and their interaction with PPIP5K2, LncOVM can aid in the infiltration of MDSCs in TME, which leads to lymphatic metastasis in ovarian cancer cells. By blocking this pathway, the C5aR antibody or inhibitor (CCX168) can prevent the recruitment of MDSCs and reinstate the *in vivo* suppression of tumor genesis and metastasis (143). Ferroptosis inducers in conjunction with ICDs for ovarian cancer have not been investigated for their precise process and effectiveness, but this combination might represent a potent target for ovarian cancer therapy. Due to its unique way of competing with T cells for cysteine, glutamine metabolism will be targeted by MDSC to lower its activity and down-regulate the immunosuppressive tumor microenvironment at the same time. Ferroptosis inducers that affect the Xc-system could be more important in promoting immunity and preventing immunological escape.

DAMPs such as HMGB1, CRT, ATP, and PGE2 are created when ferroptosis occurs in ovarian cancer cells. These molecules primarily operate on DC through various signals; DC can also act on CD8+T cells by releasing cytokines like IL-1β, IFN-γ, IL-8, and so on. T cells can control the incidence of ferroptosis by inhibiting the Xc system through JAK/STAT1 and promoting Fe through the release of IFN-γ. Macrophages mainly influence the Xc system by means of TGF-β1. To control ferroptosis, immunosuppressive cells can operate on the GPX4 system in a variety of ways (**Figure 2**).

### 4.4 Related gene prediction models

Using bioinformation technology, several researchers have constructed prediction models for ovarian cancer and discovered ferroptosis genes that are associated with both tumor inhibition and ovarian cancer prediction. The ferroptosis driving gene ALOX12, for instance, was found to be overexpressed in ovarian cancer cells and induce lipid production, showing high sensitivity and specificity for serous ovarian cystadenocarcinoma (144). Its increased expression was associated with a poor prognosis in OC patients, according to multiple studies using the mRNA expression data of TCGA, IGCG, GTEx, and clinical OC patients. ALOX12 may be a possible risk factor for OC, as inhibition of ALOX12 decreases the migration and proliferation of ovarian cancer cells (145, 146). Comprehensive evaluation of gene expression, related signaling pathways, and immunomodulatory functions also found that the ferroptosis related gene PRNP also played a tumor suppressor role in OC. It may be a potential new biomarker for OC diagnosis, prognosis, and immunotherapy response (147).

The identification of appropriate and effective immune prediction targets will significantly increase the practicality of immunotherapy for ovarian cancer. The realization and implementation of ovarian cancer immunotherapy must take into consideration the immunosuppressive network of ovarian cancer. Researchers are increasingly developing immunotherapy bio-information technology models that utilize ferroptosis-related genes to predict the effects, side-effects, and prognosis of immunotherapies. Studies have constructed a model containing nine ferroptosis-related genes, showing that ferroptosis is closely linked to tumor immunity through the ssGSEA method, but further experimental validation is needed (141). When paired with clinical variables, the risk model developed by YE et al. based on five ferroptosis-related characteristics linked to tumor immunity can more accurately predict the prognosis of OC patients (146). A model of 15 FRGs (c) divided patients into high- and low-risk groups, showing good survival differences. Additionally, in the low-risk group, characteristic enrichment sets were detected with immunity pathways related to ovarian cancer, which suggests that the model can be used to precisely treat immunotherapy for ovarian cancer and for prognosis (148). Using Cox analysis, three prognostic genes were identified from 63 FRGs. Significant differences were found in activated DCs, plasma cells, M0 infiltration, and important immune checkpoint molecules between the two groups when the CIBERSORT algorithm was applied to the various tumor immune microenvironments between the two groups based on the grouping of prognostic genes. The low-risk group also responded better to immunotherapy and chemotherapy at the same time, which was predictive of prognosis and immune response (149). Similar outcomes were achieved by Wang et al. when they developed a risk score model based on various prognostic signal FRGS. Additionally, it has been proposed that in high-risk patients, the combination of immunity and ferroptosis may result in a worse prognosis (150). A clinical risk stratification tool based on four DEG (differentially expressed genes from immune and ferroptosis) has been developed for serous ovarian cancer. It can exhibit a strong correlation with immune markers and induced pluripotent stem cells (IPS). Encourage additional clinical judgment in the areas of personalized treatment planning, prognostic assessment, and follow-up scheduling (151). A risk model established by finding significantly differentially expressed genes associated with ferroptosis was found to have favorable immune cell and prognostic associations (152). Furthermore, ferroptosis and oxidative stress-related genes (FORGs) have been found to be associated with immunotherapy in ovarian cancer. Based on the expressive characteristics of 19 FORGs, ovarian cancer patients were divided into two FORG subtypes. The high-risk group had immunosuppression and a poor prognosis. The risk scores significantly correlated with immunosurgery expression and chemotherapy sensitivity, demonstrating the usefulness of prognostication and chemotherapy for O.C (153). Xiang et al. conducted molecular subtyping of ferroptosis-related genes in ovarian cancer and developed a predictive model. They discovered that high-risk patients exhibited a tumor immune microenvironment with increased infiltration of M2 macrophages and reduced numbers of CD8+ T cells, which impaired immune responses and led to poor prognoses (154). Li and colleagues also construct risk models in conjunction with genes related to ferroptosis and cuprotosis, which can predict individual sensitivity to various immunotherapies and chemotherapy drugs based on specific groups, and have strong immune prediction and prognostic ability (155) (**Table 3**).

**Table 3:** The role of ferroptosis related gene in prediction and immunotherapy of ovarian cancer cell.

ferroptosis-related genes	Prognostic function	Related to immune response	reference
PRNP	√	√	(147)
15-FRG (CYBB, VDAC2, SOCS1, LINC00472, ELAVL1, IFNG, IDH1; NRAS, MT1G, ACSL3, SLC3A2; PTGS2, SLC1A4, PCK2, XBP1)	√	√	(148)
CXCL11、CX3CR1、FH、DNAJB6	√	√	(151)
SLC7A11, ZFP36、TTBK2	√	√	(149)
ALOX12、ACACA、SLC7A11、FTH1、CD44	√	√	(146)
FORG	√	√	(153)
HIC1、LPCAT3、DUOX1	√	√	(150)
LPCAT3、ACSL3、CRYAB、PTGS2、ALOX12、HSBP1、SLC1A5、SLC7A11, ZEB1	√	×	(141)
CDKN1B、CXCR4、FAS、FOS、FOXO1、GABARAPL1、HDAC1、IFNG、IL24、MTMR14、NFKB1、PEX3、PPP1R15A、RB1, SIRT2	√	×	(156)
GFPT2, VSIG4, HOXA5, CXCL9, and LYPD1.	√	√	(155)
ALOX12、RB1、DNAJB6、STEAP3, SELENOS	√	×	(145)
DNAJB6、RB1、VIMP/SELENOS、STEAP3、BACH1 and ALOX12	√	×	(144)
LAMP2, NOS2, ALOX5, CD44, CHMP5, FH, GOT1, DUOX2, SLC7A11, and DDIT3	√	√	(152)
PDP1, FCGBP, EPHA4, GAS1, SLC7A11, BLOC1S1, SPOCK2, and CXCL9	√	√	(154)

√, provided; × : not provided.

## 5 Conclusion and perspectives

As a new type of cell death found to be used to suppress tumor cells, ferroptosis is significant and has recently become a hotspot in the field of tumors. The regulatory role of specific mechanisms such as the GPX4 (157) and Xc system in ferroptosis and related pathways and targets has been gradually clarified. Many new mechanisms for ferroptosis, pathways and genes have been gradually discovered, but further research is still to be undertaken. Many of the currently known treatments for ovarian cancer are highly toxic and ineffective. Studies have found that ferroptosis combination therapy can help increase ovarian cancer sensitivity to the drug and improve the prognosis. The mechanism of ferroptosis is closely related to oxidative stress and ROS production, with research often focusing on mitochondria. Ferroptosis is characterized by distinct morphological changes, including smaller mitochondria, shriveled mitochondrial membranes, and reduced or absent mitochondrial cristae, while the cell membrane remains intact and the cell nucleus size remains normal (10). In ovarian cancer, PML-PGC-1α (107) can promote mitochondrial respiration, cysteine (158) restriction affects Fe-S cluster synthesis in mitochondria, and compounds like eriodictyol (40) and SPIO-serum (40) exacerbate mitochondrial dysfunction, all of which regulate ferroptosis. Recent studies have identified Mitotic Arrest Deficient 2 Like 2 (MAD2L2), an important tumor-associated protein primarily located in ribosomes, as having a potential role in mitochondrial elongation. MAD2L2 can promote ovarian cancer proliferation and migration by inhibiting ferroptosis and is closely associated with various immune cells. However, the specific and complete mechanisms and pathways of ferroptosis in mitochondria remain unclear. Future research in this area is expected to provide a more comprehensive understanding of ferroptosis (157).Furthermore, due to the immunosuppressive nature of ovarian cancer, single immunotherapy currently has good therapeutic effects in only a fraction of ovarian cancer patients, therapy combined with other new tumor treatments is a new research trend. Ferroptosis, as a new type of treatment, can not only play a role in suppressing ovarian tumors but also induce immunotherapy. However, immunosuppression of ovarian cancer has been found to reduce this combined effect and even lead to bad side effects. The study also found that it may be possible to inhibit the immunosuppressive microenvironment of ovarian tumors by targeting immune cells, thereby helping in the combination of ferroptosis inducers and immunotherapy. The application of nanomaterials in this field has endless potential and can be targeted therapeutically, inducing ferroptosis to produce the effect of immunotherapy and play more accurate, longer-lasting, and with fewer side effects. However, the specific mechanisms are not clear enough. In recent years, considerable research has been undertaken on immuno-predictive models built from bioinformatics and ferroptosis-related genes, which may help in the research and development of targets and drugs for ovarian cancer treatment. In the future, the association of ferroptosis-related inducers and inhibitors with other treatments for ovarian cancer is still worth exploring.
